# Google Medical Update: Why Is the Search Engine Decreasing Visibility of Health and Medical Information Websites?

**DOI:** 10.3390/ijerph17041160

**Published:** 2020-02-12

**Authors:** Artur Strzelecki

**Affiliations:** Department of Informatics, University of Economics in Katowice, 40-287 Katowice, Poland; artur.strzelecki@ue.katowice.pl

**Keywords:** Google, medical update, health information websites, search visibility, search engine

## Abstract

The Google search engine answers many health and medical information queries every day. People have become used to searching for this type of information. This paper presents a study which examined the visibility of health and medical information websites. The purpose of this study was to find out why Google is decreasing the visibility of such websites and how to measure this decrease. Since August 2018, Google has been more rigorously rating these websites, since they can potentially impact people’s health. The method of the study was to collect data about the visibility of health and medical information websites in sequential time snapshots. Visibility consists of combined data of unique keywords, positions, and URL results. The sample under study was made up of 21 websites selected from 10 European countries. The findings reveal that in sequential time snapshots, search visibility decreased. The decrease was not dependent on the country or the language. The main reason why Google is decreasing the visibility of such websites is that they do not meet high ranking criteria.

## 1. Introduction

Some types of websites could potentially impact people’s future happiness, health, financial stability, or safety. Google calls such pages “your money or your life” (YMYL) pages [1]. Google recognizes five types of YMYL pages. First are shopping or financial transaction pages. These webpages allow people to make purchases, transfer money, pay bills, and so on, online (such as online stores and banking) [1]. Second are financial information pages. These webpages provide advice or information about investments, taxes, retirement planning, home purchases, paying for college, buying insurance, and so on [1]. Third are medical information pages. These webpages provide advice or information about health, drugs, specific diseases or conditions, mental health, nutrition, and so on [1]. Fourth are legal information pages. These webpages provide legal advice or information on topics such as divorce, child custody, creating a will, becoming a citizen, and so on [1]. Fifth are news articles or public/official information pages that are important in order to have an informed citizenry. These webpages include information about local/state/national government processes, policies, people, and laws, disaster response services, and government programs and social services; as well as news about important topics such as international events, business, politics, science, and technology, and so on [1]. Of course, not all news articles are necessarily considered YMYL.

In past studies, authors observed three main areas in analyzing search data on health and medical information: medical and health information websites, search engine result pages with medical and health information results, and data collected from Google Trends and/or Google Flu Trends and/or Google Cloud Healthcare application programming interface (API) (formerly Google Health).

In the years before Google Trends was released in 2010, a lot of research was done on medical and health information websites. Examples include searching for health information when studying focus groups [2], especially adolescents [3], young people [4], or parents [5], and types of health searches [6] and how they are readable [7], reliable [8], or evaluated [9]. In this period people used different search engines such as Yahoo! [10] and Google [11].

The quality of medical and health information websites is very important and has been measured by indicators such as authority, source, content, and so on [12]. The quality of health information websites can be checked by manual examination or automated tools. For manual checking, DISCERN questions and the Health on the Net Foundation Code of Conduct (HONcode) are used. DISCERN consists of 15 key questions plus an overall quality rating. Each question represents a separate quality criterion [13]. The 8-point HONcode assesses the following principles: authority, complementarity, privacy, attribution, justifiability, transparency, financial disclosure, and advertising policy [14]. One of its automated tools measures 18 technical quality criteria and measurable indicators. These criteria are in seven groups: author, source, currency, content, disclosure interactivity, and commercialization [15].

In terms of analyzing search engine results, this field is divided into two areas. First, researchers are evaluating organic results displayed by search engines with tools that are widely used today such as HONcode [13], the Journal of the American Medical Association (JAMA) benchmarks, and the DISCERN tool [14], and scales such as Flesch–Kincaid reading level and Flesch reading ease [14]. Results from Google are downloaded and evaluated [16] with regard to how they answer health questions [17], for example, from parents on neonatal intensive care [18], or on palliative care [19] or human papillomavirus vaccination [20]; and there are large systematic reviews of autoimmune diseases [21]. Some studies have examined data retrieved from the Google Planner tool [22], which contains information on the popularity and competitiveness of all queries used on Google in the last 12 months [23], or built their own Google-based search engine for mining radiology reports [24]. In addition, people are also using other search engines such as Yahoo! and Bing to search for information, especially to measure the popularity of online drug information on Bing and Yahoo! [25], to clarify medical queries on Bing [26], or to compare four search engines in terms of obtaining medical information [27]. Also, the Chinese search engine has been explored in terms of predicting the incidence of hand, foot, and mouth disease using search engine queries from Baidu [28]. Baidu is popular in China because of the general unavailability of Google in the country. In a few studies, researchers built [29], prepared [30], or used [31] specialized medical search engines and tested how the results were perceived by users.

Second, participants are invited to examine search queries and use them to find health and medical information. It has been shown that manipulating the presentation of search results for common symptoms, such as the frequency and placement of serious illness mentions within search results, can influence perceptions of symptom severity and susceptibility to having the serious illness [32]. Participants usually take part in interviews [33]. It has been observed that people without university education [34] and university freshmen [35] have to make evaluations when they are searching for health information on the Internet. Participants are observed with regard to how they solve complex health information tasks using a search engine and whether there is a difference in the amount of searches and time spent on searching among different age groups [36] or the use of specified medical search engines in comparison to Google [37]. Observed participants have a high tendency to use search engines in seeking health information, especially Google [38], however the information is not always complete and reliable [39]. Parents search for health and medical information before taking a child to the emergency department [40]. Many women play a key role in providing advice and health care for family members, by searching for health and medical information using search engines [41]. It looks like the same behaviors do not differ across different countries [42].

Finally, in recent years much research has been done using data collected from Google Trends (GT), Google Flu Trends, and Google Cloud Healthcare API. There is growing amount of research using GT [43]. Before GT was released, early studies were done on Google Flu Trends, a source for queries connected to diseases [44]. GT is a source of reverse-engineered data. It shows what was searched in Google, and the data are normalized in terms of search frequency and presented in relative search volumes. Data are segmented into years and months, and into geographical regions. Researchers can compare a maximum of five keywords using segments in one try. Studies on GT can be divided into four areas—infectious diseases, mental health, other diseases, and general population behavior [45]—and are mainly conducted to examine seasonality [46].

Looking at recent studies, GT has been used to track infectious disease data on chickenpox [47], Lyme disease [48], the Ebola epidemic [49], syphilis [50], conjunctivitis [51], and dengue fever [52]. Some researchers studied mental health [53] and depression [54] queries. Other topics studied using GT data are skin cancer [55], sunscreen use [56], sunburn [57], seasonality of bruxism [58], multiple sclerosis [59], cancer [60], stroke [61], HIV [62], lupus [63], norovirus [64], sepsis [65], pertussis [66], epistaxis [67], plague [68], rheumatoid arthritis [69], and prostate cancer [70]. In terms of general population behavior, research was done using GT data on pharmaceutical data [71], vaccinations [72], movement disorders [73], digital epidemiology [74], kidney stone surgery [75], foot and ankle pain [76], knee injuries [77], osteoarthritis [78], seasonality of cellulitis [79], tracking influenza epidemics using climate data [80], palliative care [81], cosmetic body procedures [82], and anesthesia [83]. GT is also used for forecasting [84], real-time surveillance [85], or prevention [86] of diseases.

Basic search engine visibility is combined data of unique keywords, positions, and URL results. According to the concept of search engine visibility described in [87], the visibility of websites in search engines comes from algorithms that rank and order them according to calculated ranking positions. The original concept [88] of ranking for the Google search engine is named PageRank, after one of Google’s founders. PageRank was invented and published in 1998 [89]. This concept takes into account incoming links, and based on volume and quality, ranking positions for websites and corresponding keywords are estimated [90]. Currently, web search engines use different ranking factors for websites to determine their position on a results page.

Today this topic is attracting more attention [91] and can be divided into onsite and offsite factors [92]. Onsite factors are domain-, website-, and page-related [93]. Search engines take into account different elements found in the source code of a webpage such as title, headings, descriptions, time of last update, mobile design, and structured data for rich snippets [94]. Offsite factors are link-related [95], user action-related [96], special rules-related [97], brand-related [98], and spam-related [99].

The motivation behind the present study is to analyze what types of websites experience decreasing visibility on search engine results pages due to low-quality medical and health information content. There is no doubt that much research has been done on health and medical information websites and Google as a source of medical knowledge. However, there is little knowledge of the ways medical and health information websites are lowered or removed from search engine results pages due to low-quality content.

Thus, the current gap represents a lack of research on the decreasing visibility of medical and health information websites. There have been several studies on medical and health information presented on Google, conducted mainly by researching GT. Recognizing which websites are not considered to be proper sources of medical and health information by Google and why is a gap the author is trying to fill.

The objective of this study was to analyze data from an external service that monitors Google’s search engine results pages and collects data on websites’ visibility. By measuring increased or decreased visibility of health and medical information websites, it is possible to recognize the websites that are considered to have low-quality content. Based on the above discussion, the following research questions related to decreasing visibility of health and medical information websites in Google are proposed:

1. Why is the Google search engine decreasing the visibility of health and medical information websites?

2. How can we measure the decreased visibility of health and medical information websites?

The paper is organized as follows. Section 2 includes the method, search engine visibility concept, and material for data retrieval and processing. Section 3 contains the results, while Section 4 presents the discussion. In Section 5, the author highlights the contribution of the research, discusses its limitations, and, finally, draws conclusions about the results and proposes possible future research avenues.

## 2. Materials and Methods

This study used search engine visibility data on websites with health and medical information. The author selected European countries based on criteria using this list: https://en.wikipedia.org/wiki/List_of_European_countries_by_population, including countries located in Europe, not in Asia (excluding Russia, Turkey, and Kazakhstan), and countries where Google operates. For the first to ninth positions on the list there was no doubt about population. For the tenth position, five countries had >10 million population, which was sufficient for choosing one of them. Greece was chosen, since this part of Europe was not yet represented in this study and French- and Dutch-speaking countries were already on the list, which is why Belgium was not chosen. Countries with higher populations have more Internet users, thus it is more likely that there are many health and medical websites. Ten countries were selected to check whether visibility in a search engine depends on the country or/and language or is not influenced by either.

The author analyzed the top 20 results on a sequence of keywords: “google medical update site:.cc,” where “site” is a search operator that narrows the results, in this case to country domain name, and “.cc” means country-coded domain name. The author selected 10 countries with the highest populations in Europe: Germany, France, United Kingdom, Italy, Spain, Ukraine, Poland, Romania, the Netherlands, and Greece.

The term “Google medical update” refers to changes in Google’s algorithm starting on 1 August 2018 [100]. Results for these sequences of keywords allowed for collection of websites potentially affected by decreased visibility by the Google search engine. The new algorithm rewards websites with well-researched, accurate health and medical content and decreases the visibility of those whose content is lacking in terms of credibility [100].

The top 20 results returned by Google for the query “google medical update site:cc” were examined and the author collected the list of websites as a convenient sample that could be the subject of further study. The steps for searching and examining results were repeated 10 times for each country, using the country-coded domain suffix. Table 1 shows a comprehensive list of found and selected websites for further examination.

In Table 1, Code refers to the country code used in the search query. Almost all of the websites collected use country-coded domain names; however, some websites use generic domain names such as “.com” or “.org” or others such as ”.to,“ which belongs to Tonga but in Polish means “it.” Most websites use the official language; for example, Ukrainian websites are in Russian. Index size is the number of results displayed by the Google search engine for the search operator “site:website.” Although Google displays a maximum of 1000 results, this number is shown below the query and above the first results. It is a size indicator of the website and estimated number of pages that belong to one website. Index size was retrieved on 19 December 2019.

Visibility in search engines is measured as the number of keywords, positions, and visible pages and can be used to compare competing organizations in one common area or industry [101,102]. The comparison will disclose the search market share of each compared organization [103]. Based on this comparison, further analysis of Internet strategies in marketing, sales, promotion, and publishing can be done. Visibility in search engines is always subject to algorithms that sort and set rankings of results based on type of content, metadata, and models of content creation [104,105,106].

In this study, the data did not originate from Google, but from external services. Data regarding visibility was retrieved through the commercial online tool Ahrefs [107]. This tool is specialized in retrieving and saving data about website visibility in search engines. Ahrefs, except to preserve basic visibility, imports additional data and develops its own visibility metrics. These data were used to compare search engine visibility of websites on health and medical information before and after they were affected by Google’s new update.

The method of collecting data from a search engine is called scraping. Usually search engines, in their terms of service, do not allow data scraping. However, it is impossible for search engines to differentiate scraping, when done very gently, from normal user search behavior. Users use search engines dozens of times a day, and only if the search engine recognizes different traffic from the user’s network can it ask for a CAPTCHA (Completely Automated Public Turing test to tell Computers and Humans Apart) to solve, to prove that entered queries are not automated. Google does not share any download or export methods for results or provide an API for exporting search results. The only way to obtain data is to scrape them directly from the results. Scraping Google is against the terms of service. Online tools such as Ahrefs allow subscribed users to use scraped data. These tools use scraping on a large scale to obtain data from Google. The next section shows results from the visibility of the websites examined.

## 3. Results

The first step of the study was to collect data on the visibility of health and medical information websites. The list contains 21 websites that are popular in the 10 most populated European countries. Using Ahrefs, four data snapshots were retrieved. Each snapshot has a 5- or 6-month time interval. The collected data were run through a search visibility metric developed by Ahrefs, built on the number of keywords and positions and estimated click-through ratio. Data snapshots were taken with the following timestamps:

- Snapshot S1: 30 July 2018
- Snapshot S2: 1 January 2019
- Snapshot S3: 1 June 2019
- Snapshot S4: 30 November 2019

The visibility metric estimates the total monthly search traffic to the target website from the organic search results. It is calculated as the sum of traffic from all keywords for which the target website ranks in the search engine results page. The data retrieved are presented in Table 2.

The data retrieved have very large scope, which strongly depends on the index size in the Google search engine. Websites with more indexed pages have more chances to be visible in search results, because more particular webpages can be displayed. Visibility strongly depends on keywords, resulting in webpages being shown in search results. The more pages indexed from one website, the more keywords will result in a search engine results page.

**Figure 1:**
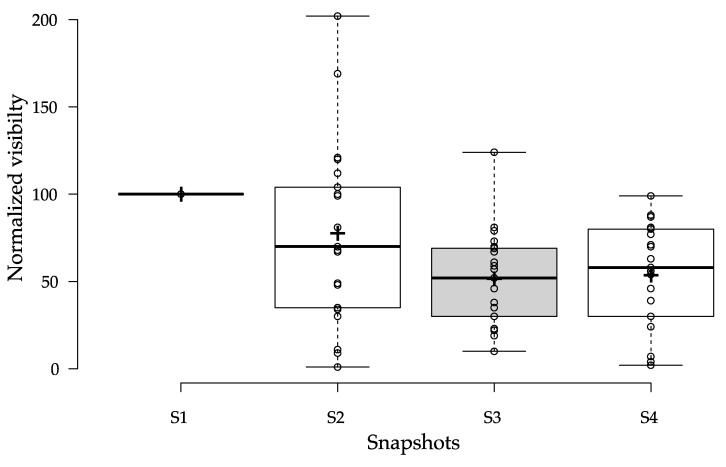
Boxplot presenting relative visibility values for 21 websites in snapshots.

In the second step of the study, data were normalized in a similar way to data presented in Google Trends. In GT, the most frequent keyword is set to a score of 100 and is used as an indicator. Other keywords are relative to this indicator and have scores between 1 and 100. In this dataset, all results for snapshot S1 were normalized to 100 and were used as the starting visibility indicator. Then, results from the next three snapshots were relative to the starting indicator. Results from four snapshots are presented in Figure 1.

Figure 1 is a boxplot illustrating that visibility decreased in the following time snapshots. Snapshot S1 was taken two days before Google announced changes in its algorithm for health and medical information websites. The value for each website was normalized to 100, which is why all descriptive statistics in Table 3 for snapshot S1 equal 100. Data from the second snapshot reveal that changes in Google’s algorithm were observed. In snapshot S2, five websites had increased visibility, one had the same, and 15 websites had decreased visibility in Google search engine results. Descriptive statistics for snapshot S2 show a median of 70 and mean of 77.57, whereas previously both were 100.

In snapshot S3, decreased visibility is still observable. Only one website had higher visibility compared with the starting date. Other websites measured had further decreased visibility in Google search engine results. Descriptive statistics for snapshot S3 show a median of 52 and mean of 51.43. In snapshot S5, visibility stayed on the same level as in the previous timestamp. Parts of websites have better visibility than in S3, but the dataset still had lower visibility compared with the starting date.

Table 3 presents descriptive boxplot statistics for all snapshots. It shows that visibility in the observed periods changed, and in this dataset, visibility decreased in snapshots S2 and S3. The last snapshot, S4, is very similar to the previous one.

It was stressed in Section 2 that visibility over a long period of time depends on many factors. Search engines take into account different factors found inside and outside websites and treat them as ranking signals. All of these factors over a long period of time influence websites’ visibility. In a shorter period of time, large changes in visibility are the effects of changes in Google’s ranking algorithm. This proves that the studied websites had decreased visibility after Google rolled out its medical update.

## 4. Discussion

The main finding of this study is that websites that did not meet high ranking criteria in terms of health and medical information were lowered in ranking since 1 September 2018. According to Google’s general guidelines, the search engine considers three areas of a website when rating the quality of a page [1]. The first is webpage content, by identifying main content, supplementary content, and advertisements. The second is understanding the website by finding the homepage, who is responsible for the website, and who created the page content, and finding sections on the page such as “about us,” contact information, or customer service information. The third is evaluating the reputation of the website or the creator of the main content by identifying sources of information on reputation and customer reviews of businesses. Page quality rating is based on how well the page achieves its purpose.

According to the results of this paper, the websites studied have lower visibility in the Google search engine. Since the exact criteria used by Google are not generally known (e.g., the current ranking algorithm is considered confidential), it is assumed that the main reason for the lower visibility is low-quality content. Low-quality websites may have been intended to serve a beneficial purpose. However, they do not achieve their purpose well because they lack an important dimension, such as having an unsatisfactory amount of main content, or because the creator of the main content lacks expertise for the purpose of the website.

The observed change in Google’s algorithm is about health and medical information websites. Until this change, this topic was unregulated. As the answer to research question 1, the author found that anyone can create health and medical content and publish it online. It does not need to be checked and corrected by a medical professional. Many people search for health and medical information using Google, and researchers use data on this from GT. However, content created without any professional supervision can be misleading and ultimately dangerous. Inaccurate information can cause unforeseen consequences such not visiting a doctor or having a false sense of security. That is why the most accurate, respected, and thoroughly researched health and medical content is displayed at the top of the search engine results.

To measure decreased visibility, it is necessary to have a sample of websites. All websites need to be measured at the same time with the same visibility metric. In this study, the author used Ahrefs data as the source of visibility. When sequential data snapshots reveal that calculated metrics are decreasing in each timestamp, it proves that visibility is decreasing. This was observed for 21 websites collected as a convenient sample for this study. The results show also that visibility does not depend on the country or language of the website, thus answering research question 2 as well.

To the best of the author’s knowledge, this is the first study to use data on search visibility on Google to assess the fluctuation of health and medical information websites in search engine results. Moreover, this is one of the first studies to compare Google visibility data between multiple countries and languages.

## 5. Conclusions

Google has very high page quality rating standards for YMYL pages, because low-quality YMYL pages could potentially have a negative impact on users’ happiness, health, financial stability, or safety [1]. In terms of medical and health information websites, medical advice should be written or produced by people or organizations with appropriate medical expertise or accreditation. Medical advice or information should be written or produced in a professional style and should be edited, reviewed, and updated on a regular basis.

It is possible to have everyday expertise in YMYL topics. For example, there are forums and support pages for people with specific diseases. Sharing personal experience is a form of everyday expertise. If forum participants tell others how long their loved ones lived with liver cancer, this is an example of sharing personal experience (in which they are experts), not medical advice. Specific medical information and advice (rather than descriptions of life experiences) should come from doctors or other health professionals. Formal expertise is important for topics such as health and medicine.

The strength of this work is in pointing out that the dominant search engine has started to rate health and medical information websites more rigorously than before. This approach can be observed using the method proposed in this work. The weakness of this work is that low quality was only assumed in manually examining these websites. Most of them offer pseudo therapies or health tips not sustained by scientific evidence, and even cooperate to provide a platform for distributing fake health tips.

This study has several limitations. First is that the observation was conducted in only one area. It does not reflect other types of information-centred websites under higher page quality ratings, such as financial, legal, or government sites. Two health and information websites from each country were the subject of the study; however, this sample size cannot adequately represent the whole area. To make the conclusions more convincing, data from more websites will be collected in the future. Second, the observation was conducted only for 10 European countries. This observation does not reflect online health and information websites in other countries globally. Data reflecting more countries will be collected in order to further investigate the role of Google’s medical update in online health and information websites. Third, although each health and information website was observed in terms of the same factors, there are still unobservable factors such as brand recognition across online webpages, which might influence their search visibility. Further studies will retrieve more data to address this issue.

One avenue of future research is to study how health and information websites are reacting to decreased visibility and measures they take to counteract this decrease. Another direction for future research is to study health and information websites for which visibility has increased and analyze which factors influenced the increase.

## Figures and Tables

**Table 1:** List of collected websites across 10 European countries for Google medical update.

Country	Code	Website	Language	Index Size
Germany	de	bessergesundleben.de	German	9260
Germany	de	gesundheitsberater-berlin.de	German	7730
France	fr	docteurclic.com	French	8310
France	fr	amelioretasante.com	French	11,000
United Kingdom	uk	bmihealthcare.co.uk	English	15,500
United Kingdom	uk	theprivateclinic.co.uk	English	2790
Italy	it	pazienti.it	Italian	85,400
Italy	it	farmacoecura.it	Italian	5400
Spain	es	reproduccionasistida.org	Spanish	29,400
Spain	es	lavidalucida.com	Spanish	7130
Ukraine	ua	doc.ua	Russian	84,200
Ukraine	ua	likarni.com	Russian	89,300
Poland	pl	poradnikzdrowie.pl	Polish	127,000
Poland	pl	portal.abczdrowie.pl ^1^	Polish	180,000
Poland	pl	wylecz.to	Polish	23,500
Romania	ro	csid.ro	Romanian	67,300
Romania	ro	sfatulmedicului.ro	Romanian	461,000
Netherlands	nl	ziektevrijleven.nl	Dutch	271
Netherlands	nl	boerenmedical.nl	Dutch	1820
Greece	gr	healthyliving.gr	Greek	28,600
Greece	gr	medlabgr.blogspot.com ^1^	Greek	13,300

^1^ Index size is measured for a subdomain.

**Table 2:** Visibility data of 21 websites considered as decreased by Google medical update.

Website	S1	S2	S3	S4
bessergesundleben.de	399,758	140,437	139,965	14,760
gesundheitsberater-berlin.de	45,200	21,670	20,603	28,465
docteurclic.com	712,711	711,744	154,889	568,476
amelioretasante.com	1,215,983	17,454	231,230	84,006
bmihealthcare.co.uk	93,293	62,312	65,098	66,319
theprivateclinic.co.uk	31,597	10,882	24,845	9584
pazienti.it	1,515,014	1,691,641	867,826	694,602
farmacoecura.it	3,008,684	3,608,904	2,434,143	2,967,836
reproduccionasistida.org	643,038	55,762	62,037	12,390
lavidalucida.com	383,920	40,333	74,143	6100
doc.ua	266,285	80,954	182,940	187,664
likarni.com	143,864	143,138	87,398	125,360
poradnikzdrowie.pl	12,592,643	13,130,013	4,821,490	9,730,085
portal.abczdrowie.pl ^1^	6,110,596	3,018,926	3,588,311	1,489,164
wylecz.to	1,990,617	2,401,242	1,337,807	1,146,077
csid.ro	1,894,383	3,205,719	438,859	1,655,861
sfatulmedicului.ro	1,709,594	1,165,206	516,015	954,856
ziektevrijleven.nl	5669	4605	2960	2219
boerenmedical.nl	7718	7620	9544	6229
healthyliving.gr	183,773	371,273	134,460	162,141
medlabgr.blogspot.com ^1^	157,154	110,688	72,118	85,238

^1^ Visibility is measured for a subdomain.

**Table 3:** Boxplot statistics for Figure 1.

	S1 ^1^	S2	S3	S4
Upper whisker	100	202	124	99
3rd quartile	100	104	69	80
Median	100	70	52	58
1st quartile	100	35	30	30
Lower whisker	100	1	10	2
No. of data points	21	21	21	21
Mean	100	77.57	51.43	53.57

^1^ Snapshot S1 is normalized to 100.
